# Evaluation of Intra- and Inter-Network Connectivity within Major Brain Networks in Drug-Resistant Depression Using rs-fMRI

**DOI:** 10.3390/jcm13185507

**Published:** 2024-09-18

**Authors:** Weronika Machaj, Przemysław Podgórski, Julian Maciaszek, Patryk Piotrowski, Dorota Szcześniak, Adrian Korbecki, Joanna Rymaszewska, Anna Zimny

**Affiliations:** 1 Department of General and Interventional Radiology and Neuroradiology, Wroclaw Medical University, Borowska 213, 50-556 Wroclaw, Poland; 2 Department of Psychiatry, Wroclaw Medical University, Pasteura 10, 50-367 Wroclaw, Poland; 3 Department of Clinical Neuroscience, Faculty of Medicine, Wroclaw University of Science and Technology, WUST Hoene-Wrońskiego 13c, 50-372 Wroclaw, Poland

**Keywords:** resting-state functional MRI, cortical activity, functional connectivity, major depressive disorder

## Abstract

**Background:** Major Depressive Disorder (MDD) is a significant challenge in modern medicine due to its unclear underlying causes. Brain network dysfunction is believed to play a key role in its pathophysiology. Resting-state functional MRI (rs-fMRI), a neuroimaging technique, enables the in vivo assessment of functional connectivity (FC) between brain regions, offering insights into these network dysfunctions. The aim of this study was to evaluate abnormalities in FC within major brain networks in patients with drug-resistant MDD. **Methods:** The study group consisted of 26 patients with drug-resistant MDD and an age-matched control group (CG) of 26 healthy subjects. The rs-fMRI studies were performed on a 3T MR scanner (Philips, Ingenia) using a 32-channel head and neck coil. Imaging data were statistically analyzed, focusing on the intra- and inter-network FC of the following networks: default mode (DMN), sensorimotor (SMN), visual (VN), salience (SN), cerebellar (CN), dorsal attention (DAN), language (LN), and frontoparietal (FPN). **Results:** In patients with MDD, the intra-network analysis showed significantly decreased FC between nodes within VN compared to CG. In contrast, the inter-network analysis showed significantly increased FC between nodes from VN and SN or VN and DAN compared to CG. Decreased FC was found between SN and CN or SN and FPN as well as VN and DAN nodes compared to CG. **Conclusions:** Patients with MDD showed significant abnormalities in resting-state cortical activity, mainly regarding inter-network functional connectivity. These results contribute to the knowledge on the pathomechanism of MDD and may also be useful for developing new treatments.

## 1. Introduction

Major Depressive Disorder (MDD) is a widely prevalent mental health condition. The World Health Organization (WHO) anticipates that by 2030, depression will be acknowledged as a significant global burden [1]. The primary symptoms of MDD include persistent depressed mood as well as impaired motivation and cognitive function. These symptoms often lead to difficulties in daily activities, a progressive loss of interest in previously enjoyed activities, and, in severe cases, complete withdrawal from them [2,3,4,5,6]. Diagnosis mainly relies on clinical evaluation, supplemented by biochemical, genetic, or brain imaging studies. Currently, most therapies focus on pharmacological enhancement of neurotransmitter levels. However, the individual response to the treatment may vary, and drug resistance is becoming more frequent among patients with MDD [7]. Recently, newer treatment modalities, such as deep brain stimulation (DBS) and transcranial magnetic stimulation (TMS), have been developed for patients who do not respond to conventional treatments [8].

The pathophysiology of MDD is complex and not fully understood, with multiple theoretical models attempting to explain its underlying mechanisms. The monoamine deficiency hypothesis posits that a lack of neurotransmission involving serotonin or catecholamines contributes to MDD’s onset. This hypothesis gained support because medications that inhibit monoamine oxidase (MAO) and thereby increase the availability of these neurotransmitters have been shown to alleviate depressive symptoms. However, this explanation has been increasingly questioned, particularly because there is often a delay of several weeks before the therapeutic effects of antidepressants become apparent. If MDD were purely a result of such an imbalance, the mood improvements from antidepressants would be expected to occur much more rapidly, given their immediate effects on neurotransmitter levels at synapses [9,10,11]. This discrepancy has led researchers to explore alternative explanations, focusing on more intricate mechanisms involving the brain’s structural and functional organization. An emerging view suggests that MDD is not merely the result of altered neurotransmitter levels but is also linked to pathological changes in neuronal connectivity and dysfunctional information processing within brain networks. This theory is supported by modern brain imaging studies, which reveal widespread disruptions in connectivity between key brain regions involved in mood regulation, cognition, and emotion processing in individuals with MDD. The concept of pathological neuronal connectivity is now central to many etiological models of MDD, reinforced by evidence that antidepressants may help reorganize these dysfunctional connections [8,10,11,12,13,14,15,16,17,18].

Resting-state functional magnetic resonance imaging (rs-fMRI) is an advanced MR technique that enables the in vivo assessment of cortical activity and brain network connectivity. It has become widely used in the study of many psychiatric disorders, including MDD. Rs-fMRI is particularly beneficial for studying MDD because it captures the brain’s intrinsic connectivity without requiring task-related activation, which can be influenced by mood fluctuations or variability in task performance—factors commonly observed in individuals with MDD. This technique allows researchers to examine spontaneous neural activity and connectivity patterns that are disrupted in MDD, offering deeper insights into the underlying network dysfunctions that may not be as evident in task-based paradigms. Moreover, rs-fMRI reduces the cognitive and motivational demands on patients, who may struggle to engage in specific tasks during scanning. This results in more reliable and representative data, making it especially effective for studying large-scale brain networks and their interactions in a natural resting state. Thus, rs-fMRI provides a more comprehensive understanding of the functional connectivity impairments associated with MDD [19,20,21].

Previous studies have already indicated that resting-state cortex activation is altered in MDD, mainly affecting the orbitomedial prefrontal cortex, the amygdala, the hippocampus, the basal ganglia, the anterior cingulate cortex, and even the cerebellum and brainstem [8]. Previous studies focused mostly on assessing changes in one of the brain networks or changes within a specific part of the brain. They mostly used the seed ROI method to evaluate the functional connectivity (FC) between a certain brain region or subregion (seed) and other brain areas. Nevertheless, their results are often ambiguous, sometimes even contradictory. Moreover, the research methods, study protocols, and even the scanners used in the various centers have been very inconsistent, which makes the results very difficult to compare between the studies [8,19,22,23,24,25,26].

Based on many previous reports on resting-state connectivity in MDD, it appears that mood-related disturbances are not attributed to a single brain area or even a few regions. Instead, it is the dysconnectivity across multiple areas, and ultimately the entire brain, that contributes to the development of mood disorders [8,27]. MDD modifies the brain system profoundly, but still little is known about specific patterns of the network-level disorganization.

Our study aimed to evaluate differences in functional connectivity (FC) both within and between eight major brain networks—default mode network (DMN), dorsal attention network (DAN), cerebellar network (CN), salience network (SN), sensorimotor network (SMN), frontoparietal network (FPN), language network (LN), and visual network (VN)—in patients with Major Depressive Disorder (MDD). We hypothesized that significant differences in functional connectivity (FC) would be found between individuals with MDD and healthy controls. The null hypothesis posits no significant differences in FC between the two groups. The primary endpoint of this study was to assess changes in FC both within and between the eight major brain networks, while secondary endpoints focused on identifying specific networks or connections most affected in MDD. Using an ROI-to-ROI approach, we assessed FC across multiple brain networks, providing insights into global functional dysconnectivity in MDD rather than focusing on a single network or region.

To our knowledge, this is the first study using an ROI-to-ROI approach to assess network-level abnormalities in several brain networks at rest in patients with drug-resistant MDD.

## 2. Materials and Methods

### 2.1. Study Participants

A total of 26 patients with clinically diagnosed drug-resistant MDD and 26 healthy subjects (CG) matched for age, sex, and educational level were included in this study.

MDD patients were enrolled from an outpatient clinic, diagnosed according to ICD-10 criteria, and clinically assessed using the Hamilton Depression Scale (HAM-D) and the Montgomery–Asberg Depression Scale (MADRS) (Table 1). All patients had been suffering from depression for at least one year, and all had been taking antidepressants for their treatment with no improvement. Treatment resistance was established based on lack of remission in MDD symptoms despite 4–6 weeks of pharmacological therapy with serotonin norepinephrine reuptake inhibitors, selective serotonin reuptake inhibitors, or tricyclic antidepressants in adequate dosages.

This study was conducted under the guidance and approval of the Bioethical Committee at Wroclaw Medical University (KB-400/2018/2506), date of approval 25 June 2018. Each participant signed an informed consent prior to inclusion in this study.

### 2.2. Neuroimaging

Neuroimaging data were collected during a 15 min scan using a 3T MRI scanner (Ingenia, Philips, Best, Netherlands) equipped with 45 mT/m, 200 T/m/s gradients, and a 32-channel head coil. The MR protocol included a high-resolution T1-weighted sagittal sequence (number of slices = 257; repetition time (TR) = 11 ms; echo time (TE) = 5 ms; flip angle = 8°; field of view (FOV) = 256 mm × 256 mm; voxel size = 0.75 mm × 0.75 mm) and a multi-band EPI sequence (MB = 6, TR/TE = 1100/31 ms, resolution = 2.5 × 2.5 × 2.5 mm^3^). Foam padding was used to minimize head movement, and patients wore headphones to reduce noise. They were instructed to lie still, remain awake, and keep their eyes closed during the scan.

Structural and functional images were analyzed using CONN toolbox 20.b, a Matlab/SPM-based software. A standard pipeline in Statistical Parametric Mapping software was used to preprocess the data (SPM12, Wellcome Department of Cognitive Neurology, University College London, London, UK) running Matlab Release 2019b (The MathWorks, Inc., Natick, MA, USA) and Linux HPC Server running Ubuntu 18.04.

The preprocessing techniques employed slice-timing correction, realignment, segmentation, normalization to the MNI template, and smoothing. The functional data were aligned using SPM12 and the realign and unwarp procedure [28,29]. The scans were aligned and resized to match a reference image using b-spline interpolation. The SPM12 slice-timing correction procedure was used to correct for temporal misalignment between different slices of the functional data. The Artefact Detection Tools (ART) toolbox (https://www.nitrc.org/projects/artifact_detect/, accessed on 13 August 2024) was utilized to identify outlier scans [30]. Outliers were detected by analyzing the global BOLD signal and measuring the level of subject motion during scanning. Outliers were defined as scans with deviations exceeding three standard deviations from the global mean BOLD signal or with a framewise displacement greater than 0.5 mm.

Next, both functional and anatomical scans were normalized to standard MNI space, with a final voxel size of 1 mm, and segmented into gray matter, white matter, and cerebrospinal fluid using SPM12’s unified segmentation and normalization procedure [31]. To reduce confounding factors in the resting-state fMRI (rs-fMRI) data, regression techniques were employed to remove signals from cerebrospinal fluid and white matter, while motion correction procedures were implemented to account for head movements. A high-pass filter was applied to eliminate low-frequency drifts, ensuring the BOLD signals analyzed were as free from non-neuronal influences as possible. The direct normalization procedure employed distinct segmentation and normalization techniques on both the functional and structural data. The mean BOLD signal was used as the reference image for the functional data, while the raw T1-weighted volume served as the reference image for the structural data. Both anatomical and functional data were resampled to a default bounding box of 180 mm × 216 mm × 180 mm, with 2 mm isotropic voxels for functional data and 1 mm for anatomical data, using fourth-order spline interpolation. To mitigate the effects of motion and physiological noise, the CompCor method was applied to identify and remove confounding factors from the blood-oxygen-level-dependent (BOLD) signal [32]. The fMRI data also underwent bandpass filtering between 0.008 and 0.09 Hz, using a discrete cosine transform to minimize border effects and isolate slow-frequency fluctuations [33].

To further reduce noise, multiple regression was used to remove signal contributions from cerebrospinal fluid, white matter, and head micro-movements (including three translation and three rotation parameters plus their first-order derivatives). Finally, to enhance the BOLD signal-to-noise ratio and reduce variability in functional and gyral anatomy between subjects, the functional data underwent spatial smoothing with an 8 mm full width at half maximum (FWHM) Gaussian kernel [34,35].

#### 2.2.1. First-Level Analysis

**Figure 1:**
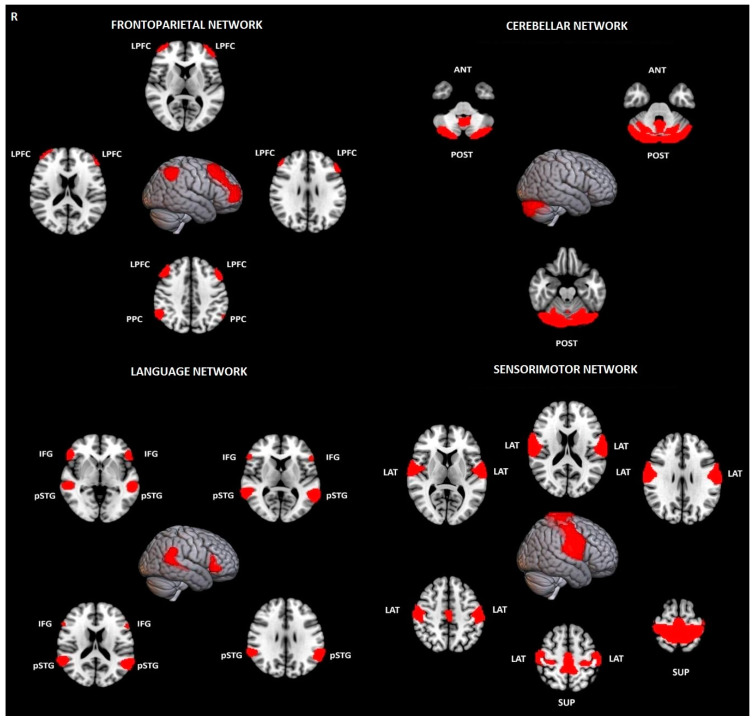
Visualization of evaluated brain networks with their nodes used as ROIs in ROI-to-ROI analysis of Evaluation of Intra- and Inter-Network Connectivity within Major Brain Networks in Drug-Resistant Depression Using. The upper row shows the frontoparietal network (FPN) and the cerebellar network (CN), while the lower row displays the language network (LN) and the sensorimotor network (SMN). Abbreviations: LPFC—Left frontoparietal cortex, PPC—Posterior parietal cortex, POST—posterior, ANT—anterior, IFG—Inferior Frontal Gyrus, pSTG—posterior Superior Temporal Gyrus, LAT—lateral, SUP—superior.

**Figure 2:**
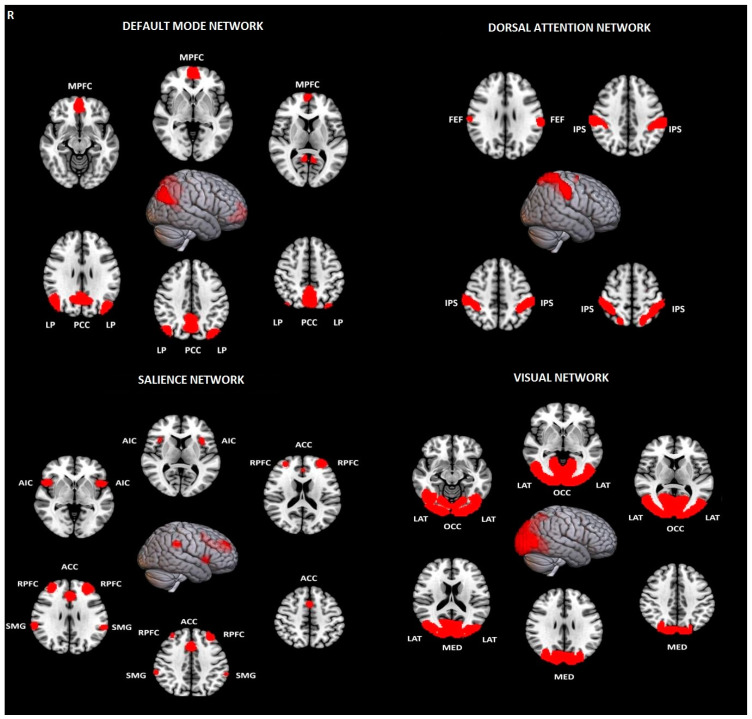
Visualization of evaluated brain networks with their nodes used as ROIs in ROI-to-ROI analysis of rs-fMRI. The top row shows the default mode network (DMN) and the dorsal attention network (DAN), while the bottom row depicts the salience network (SN) and the visual network (VN). Abbreviations: MPFC—medial prefrontal cortex, LP—Lateral Parietal, PCC—Posterior Cingulate Cortex, FEF—Frontal Eye field, IPS—Intraparietal sulcus, AIC—Anterior Insular Cortex, RPFC—Rostral prefrontal cortex, ACC—anterior cingulate cortex, SMG—Supramarginal gyrus, OCC—occipital, MED—medial, R—right, LAT—lateral.

Thirty-two region-of-interest (ROI) seeds, with predetermined shapes and locations obtained from the Human Connectome Project (HCP) atlas and adjusted to each volume, were utilized to evaluate eight resting-state networks, including the default mode network (DMN), salience network (SN), frontoparietal network (FPN), dorsal attention network (DAN), sensorimotor network (SMN), language network (LN), visual network (VN), and cerebellar network (CN) (Figure 1 and Figure 2) [36].

In this study, we utilized the nomenclature from the HCP atlas to ensure consistency and facilitate potential future comparisons. Nevertheless, we are aware of the newly suggested terminology for functional brain networks in which the default mode network (DMN) is referred to as the medial frontoparietal network (M-FPN), the salience network (SN) as the midcingulo-insular network (M-CIN), the frontoparietal network (FPN) as the left frontoparietal network (L-FPN), the dorsal attention network (DAN) as the dorsal frontoparietal network (D-FPN), the sensorimotor network (SMN) as the pericentral network (PN), and the visual network (VN) as the occipital network (ON) [37].

The method for selecting ROIs in the HCP atlas is based on an advanced, multi-modal parcellation approach that integrates data from various imaging techniques, including structural MRI, rs-fMRI, task-based fMRI, and diffusion MRI. In the HCP atlas, ROIs are defined using resting-state functional connectivity analysis, which identifies temporal correlations in BOLD signals between different brain regions. Additionally, structural connectivity derived from diffusion MRI is considered to further refine the ROIs. The final ROIs are generated through a consensus process using data from multiple participants, ensuring that they are both representative and reproducible across individuals.

Functional connectivity measures were calculated by computing bivariate Pearson’s correlation coefficients between the mean BOLD signal time courses of each pair of pre-defined seed regions in rs-networks. Sex and age were used as covariates to control for potential confounding effects. This was carried out to identify patterns of connectivity between regions of interest (ROIs). The obtained coefficients were transformed into normally distributed scores using Fisher’s transformation to better meet the assumption of normality for subsequent second-level analyses. This transformation ensures that the correlation values are more appropriately distributed for statistical testing and comparison in higher-level analyses.

#### 2.2.2. Second-Level Analysis

A second-level general linear model was employed to enable comparisons between groups. Functional connectivity (FC) scans of patients with MDD and CG were then analyzed using two-tailed paired *t*-tests. Results were considered significant only if they survived a false discovery rate (FDR) correction with a *p*-value threshold of <0.05 at the seed level.

## 3. Results

In our study, the intra-network analysis showed significantly decreased FC between nodes within VN in patients with MDD compared to CG. Reduced FC was found between the occipital and lateral nodes of VN, as well as between the lateral left and lateral right nodes of VN.

In the intra-network analysis, no additional significant functional alterations were found within other analyzed networks, such as DMN, DAN, CN, SN, SMN, FPN, or LN.

On the other hand, in patients with MDD, the inter-network analysis revealed VN nodes to show mainly significantly increased FC with SN nodes and DAN nodes compared to CG. Increased FC was found between the occipital node of VN and ACC of SN and between the medial nodes of VN and nodes of SN such as both insulae, ACC, and RPFC. On the other hand, the lateral nodes of VN showed increased FC with all nodes of SN and a single node from DAN (IPS). The occipital node of VN showed decreased FC with a single DAN node (IPS).

The inter-network analysis also revealed SN nodes to have significantly decreased functional connectivity with anterior and posterior nodes of CN and with nodes of FPN such as the bilateral LPFC and right PPC.

**Figure 3:**
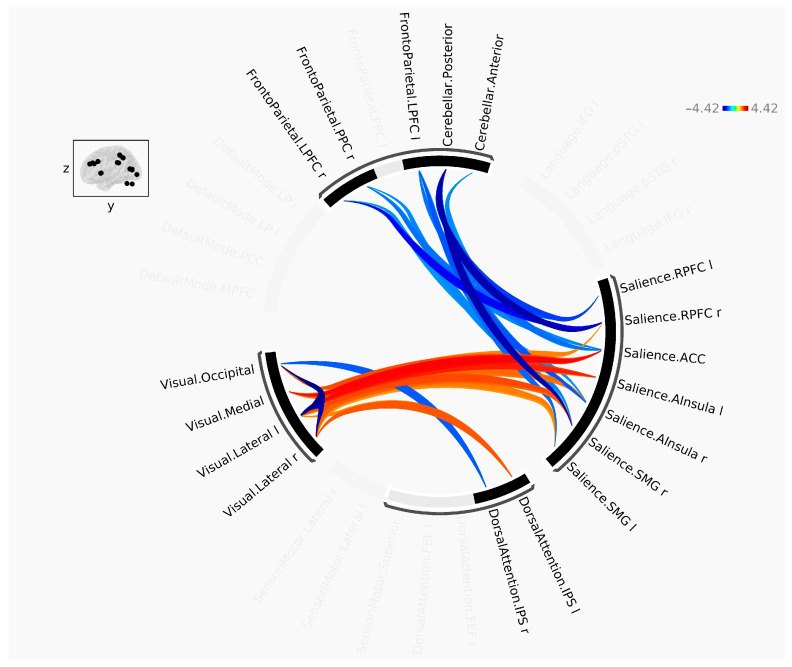
Connectome ring showing significantly increased (red) or decreased (blue) functional connectivity between different nodes of the same network (intra-network analysis) or of different networks (inter-network analysis).

**Figure 4:**
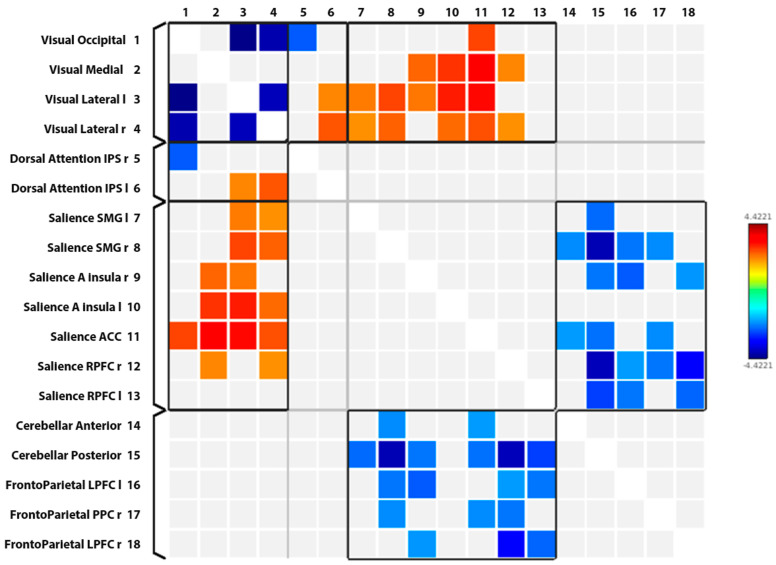
Connectome matrix showing significantly increased (red) or decreased (blue) functional connectivity between different nodes of the same network (intra-network analysis) or of different networks (inter-network analysis).

**Figure 5:**
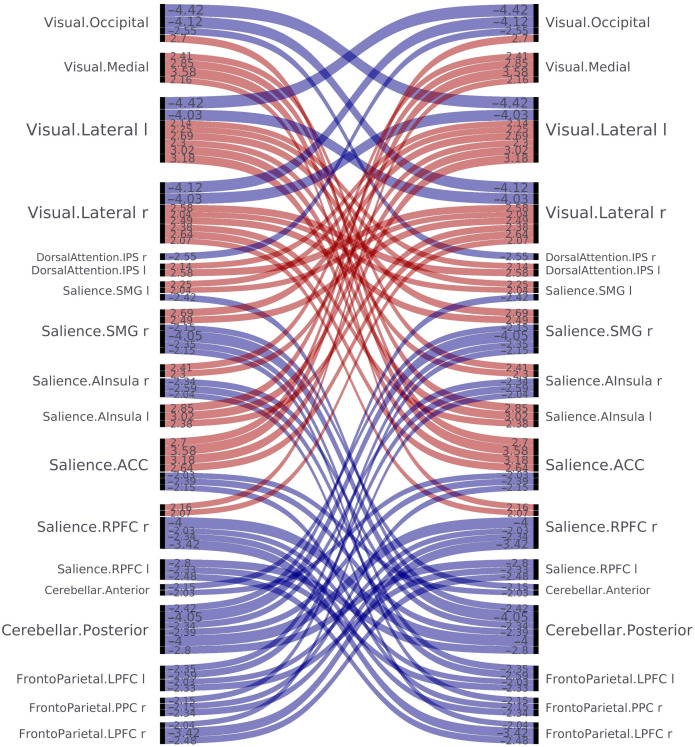
The graphical representation of the correlation coefficients illustrates the strength and direction of the connections between the analyzed regions using a color-coded heatmap. It highlights increased (red) or decreased (blue) functional connectivity, either between nodes within the same network (intra-network analysis) or across different networks (inter-network analysis).

The statistically significant results of the rs-fMRI study are presented graphically in the connectome ring in Figure 3 and the connectome matrix in Figure 4. The summarized results of the intra- and inter-network analysis are shown in Table 2, respectively (Table 2). Moreover, the correlation coefficients are graphically represented in Figure 5, providing a clear visualization of the relationships between variables (Figure 5).

## 4. Discussion

The aim of our study was to assess the functional connectivity changes within several brain networks and also between different brain networks to obtain global knowledge on brain functional changes in patients with drug-resistant MDD. We also used a novel methodological approach using an ROI-to-ROI method of rs-fMRI data analysis.

In our study, the intra-network analysis revealed alterations exclusively within the visual network (VN), with no detectable changes in the other analyzed brain networks. Functional connectivity was significantly decreased within the VN, particularly between the occipital and both lateral nodes, as well as between the right and left lateral nodes of this network. Visual system alterations in patients with MDD have long been of interest to researchers [38,39].

The advancements in fMRI technology have enabled the identification of cortical areas involved in visual function and their evaluation [40,41,42,43,44].

The spontaneous activity within the VN in healthy subjects in the rs-fMRI is possibly related to visual memory consolidation processes but also memory-related mental imagery. Additionally, the VN might play a role in the mechanism of recreating previous visual information and presenting it as mental images [45,46,47]. In the literature, there have not been many reports on VN changes in functional connectivity in patients with MDD. Results consistent with our study were obtained by Lu et al. [48]. They assessed intrinsic connectivity within visual networks by applying independent component analysis (ICA) to fMRI data collected during the resting state. Their findings indicated that MDD patients exhibited hypoconnectivity in the right calcarine (visual component 2) and the right inferior/middle occipital gyrus (visual component 3). These regions are involved in processing visual impressions, responding to stimuli, and controlling attention. Reduced connectivity in these areas can affect the brain’s overall ability to respond to environmental stimuli, which in turn influences emotional experiences and responses to various situations [48].

Moreover, a recent large-scale study by Long et al. identified significant changes in VN connectivity among MDD patients across different age groups. This study utilized rs-fMRI to examine MDD-related abnormalities in functional connectivity (FC) patterns and found a general decrease in FC within the VN among MDD patients. Notably, this reduction was most pronounced in connections between the VN and the subcortical regions, highlighting the vulnerability of visual processing pathways in depression. These disruptions were particularly significant in early- and middle-aged adults, suggesting that MDD’s impact on the VN is more pronounced during these life stages [49].

On the other hand, Chen et al. have demonstrated different results in patients with MDD using rs-fMRI and an ROI-to-ROI approach [50]. The authors have shown ventral and dorsal visual networks to exhibit significantly increased clustering coefficients, small-worldness, and also mean variability of dynamic functional connectivity. In the studied networks, local coherence was increased and, at the same time, the efficiency of long-distance information transfer was maintained and functional connectivity between the studied nodes fluctuated more than in healthy individuals [50]. These findings may suggest that, in MDD, there are dynamic changes in connectivity within the VN. These alterations do not necessarily manifest as a steady reduction in connections but rather as increased variability over time.

AbouElseoud et al. also conducted a study investigating changes in visual areas. They utilized rs-fMRI to evaluate functional network connectivity through dual ICA regression, as well as the amplitude of low-frequency fluctuations (ALFF), in patients diagnosed with seasonal depression. They identified differences in functional networks primarily located within sensory regions. Specifically, higher amplitudes of low frequency (ALFF) and increased FC across a wide range of visual cortice areas. The results they obtained may indicate increased neuronal activity in the brain areas analyzed but also some abnormalities in the processing of neuronal signals. In the context of seasonal depression, these findings may also be the effect of the brain’s regulatory activities in an attempt to adapt to certain conditions [51,52]. In conclusion, in patients with MDD, VN dysfunction may be responsible for concentration problems, learning difficulties, and an impaired ability to interpret visual information related to emotions (facial expressions), but further research is needed to confirm these suspicions [40].

The results of the inter-network analysis in our study showed either decreased or increased FC between different networks.

In this analysis, the VN was again shown to exhibit significantly changed FC with other networks. We revealed significantly increased FC between all VN nodes and several SN nodes such as ACC, both insulae, or RPFC, and increased FC between the lateral VN nodes and the left IPS of DAN, as well as decreased FC between the occipital VN node and the right IPS node of DAN. The results of our rs-fMRI study showing increased FC between VN and SN or DAN suggest overresponse or overactivity of VN connectivity with other networks. Both SN and DAN are networks important in the pathology of MDD [53,54,55,56,57]. The insula, which is a part of SN, may be involved in the misinterpretation of sensory information, which leads to abnormal responses [58]. Additionally, research on emotional regulation relates its impaired function to the ineffective regulation of and response to negative mood [59]. The PFC is one of the regions most consistently impaired in MDD. The RPFC is a specific part of this area, the dysfunction of which can cause difficulties in concentration, attention, and effective regulation of emotions [60,61]. The dorsal attention network has a crucial role in the regulation of attention, sensory information processing, spatial orientation, and cognitive control. Dysfunctions in this network may contribute to many of the symptoms of MDD, including concentration problems, negative thinking, and difficulties in decision-making. Therefore, understanding and studying the DAN network is important to better understand the mechanisms underlying depression and to develop more effective therapies [50,56].

In our study, we found significantly increased FC between all VN nodes and several SN nodes (ACC, both insulae, and RPFC). There are only a few reports in the available literature on connectivity disorders between VN and SN. In the study by McGlade et al., rs-fMRI with a seed-based whole-brain correlation method was used to assess functional connectivity (FC) in veterans diagnosed with both depression and PTSD. The results showed that females exhibited higher FC between the left basolateral amygdala (BLA) and the primary visual cortex (V1). In contrast, the severity of depression symptoms in males was associated with increased FC between the left BLA and the bilateral occipital lobes [62]. Another study by Colich et al. showed increased activation in the frontocingulate regions (including the dorsal anterior cingulate cortex) and the occipitoparietal region (including the lateral occipital cortex and the superior parietal lobule) in MDD when ignoring fear faces and neutral faces [63]. These results may imply that MDD patients need greater brain activation for cognitive control and visual attention than healthy individuals. In depression, FC variations between the VN and SN nodes may indicate changes in information processing and integration. Altered connectivity between these networks can affect mood and cognitive processes related to visual information [64,65]. The increased connectivity might be linked to the brain’s mechanism of recruiting additional volume to maintain function compared to healthy individuals. We propose that this heightened FC could represent a compensatory mechanism; however, it could also be attributed to neuronal excitotoxicity.

In our study, we found increased FC between the lateral VN nodes and the left IPS of DAN as well as decreased FC between the occipital VN node and the right IPS node of DAN. The work by Chen et al. also evaluated the FC between the dorsal and ventral visual pathways and DAN using the multivariate distance correlation. In both cases, FC was found to be reduced [50]. In another study, it was pointed out that MDD alters the visuo-attentional system, which involves abnormal modulation of the parietal lobe on the visual cortex and altered effective connectivity between the two. It has been known for long that people with MDD often exhibit attentional bias, in which their attention is selectively focused on negative stimuli. Additionally, cognition-related task MRI studies suggested changes in visual processing in people with depression, including variations in the perception of facial emotions or altered responses to visual stimuli [66]. The results of our study and some other reports on rs-fMRI prove that patients with MDD suffer from major visuo-attentional dysconnectivity.

Another important finding in our inter-network analysis was decreased FC between several nodes of SN (SMG, insulae, ACC, RPFC) and the anterior and posterior nodes of CN or nodes of FPN, such as the bilateral LPFC and the right PPC. Our study suggests hypoactivity or hyporesponse of SN with CN and FPN. Luking et al. also showed reduced connectivity between the amygdala and the dorsolateral prefrontal cortex (dlPFC), dorsomedial prefrontal cortex (dmPFC), and hippocampus [67]. Therefore, these results correspond to decreased connectivity between SN and both DMN and FPN and are partially in agreement with our work. Similar results have already been reported in the literature (Wei et al., Tang et al., Lu, Q. et al., and Siegle et al.). Nevertheless, due to significant differences in study designs, drawing common conclusions is very questionable [68,69,70,71]. Both SN and FPN are associated with emotion and mood regulation. In the context of depression, research suggests that alterations in the FPN may contribute to difficulties in mood regulation, as well as problems with concentration, attention, and the ability to process information, while SN dysfunction may be associated with increased reactivity to emotional stimuli, loss of interest, or sleep disturbances [72]. Decreased connectivity between the networks mentioned above could correspond to symptoms found in depression. Recently, the cerebellum has been recognized to be involved in a variety of cognitive and emotional processes. Structural and also functional alterations of this area have been observed in people with depression [73]. The assessment of FC between the cerebellum and cortical areas has been reported in the literature [74,75,76,77,78]. However, there are only a few studies on the functional connectivity between CN and SN. Feng et al. obtained results consistent with ours using seed-based FC analysis in a voxel-wise manner to investigate cerebellum–cortical functional connectivity in elderly women with depressive symptoms. They demonstrated reduced functional connectivity between the cerebellum (Lobule IX, X) and the right insula, which we categorized as reduced FC connectivity between CN and SN [79]. We hypothesize that the reduction in functional connectivity between these two networks is probably related to difficulties in processing environmental information and internal emotional states and the ability to respond appropriately to social cues and may also affect the ability to experience pleasure. However, further research is required to confirm these assumptions.

From a diagnostic perspective, altered connectivity, particularly within the VN and between networks such as the SN, FPN, and DAN, may offer valuable information for identifying network dysfunctions related to MDD. These abnormalities in the synchronization of brain networks have the potential to serve as biomarkers (helping in the early detection and diagnosis of MDD) and provide new opportunities for targeted treatment. Changes in the connections between and within these networks suggest possible targets for neuromodulatory therapies, such as repetitive transcranial magnetic stimulation (rTMS). Our findings suggest that targeting these specific network disruptions may increase treatment efficacy by directly addressing altered functional connectivity in MDD patients.

The observed changes in functional connectivity in these networks may reflect their crucial role in cognitive and emotional regulation, processes often impaired in MDD. Abnormalities in the SN and DMN, which are associated with attention and self-referential thinking, may contribute to emotional dysregulation. Meanwhile, reduced connectivity within the VN, normally involved in sensory processing, may relate to altered sensory perception in patients. In addition, increased connectivity between the VN, SN, and DAN may suggest compensatory mechanisms.

## 5. Strengths and Limitations

The rs-fMRI post-processing method used in our study is a global, standardized tool. The networks we assessed were defined based on data from the Human Connectome Project (HCP), which is publicly available. This approach ensures that our methods are reproducible and that our results can be easily compared in future studies. By evaluating eight major brain networks, we gain a comprehensive understanding of brain dysfunction in MDD.

However, our study has several important limitations. First, the sample size was relatively small, as we only recruited drug-resistant MDD patients eligible for TMS treatment, constrained by the project’s exclusion criteria. This limitation affects the generalizability of our findings and the statistical power of our analysis, making it difficult to compare our results with other forms of MDD.

Another limitation is the variability in the duration since the last use of antidepressants among participants, despite none being on medication at the time of our study. Residual effects from previous medications could influence the results, and although we accounted for this in our analyses, it remains a potential confounder. Future research should account for medication history more comprehensibly to better isolate the effects of current treatment status.

Regarding participant selection, we specifically included individuals who met the criteria for MDD without comorbid psychiatric conditions such as anxiety or substance use disorders. However, we recognize that the presence of comorbid conditions could potentially impact the results. Future studies should take a more comprehensive approach to assessing comorbid conditions to better understand their effects on the findings.

Finally, the use of the Automated Anatomical Labeling (AAL) atlas in our study may also pose a limitation. Automated labeling processes can introduce errors due to limitations in flexibility and spatial resolution, potentially leading to the mislabeling of brain regions. The AAL atlas does not account for individual anatomical differences within the study population either. Future research should consider using more adaptable methods that can accommodate individual variability.

## 6. Conclusions

In summary, our study found that intra- and inter-network brain functional connectivity was significantly altered in patients with MDD compared to healthy individuals. Functional connectivity within the visual network was significantly reduced, while the analysis of inter-network connectivity showed decreased FC between SN and the CN and FPN nodes, as well as VN and DAN, and increased FC between VN and both SN and DAN. Our results suggest that changes in the synchronization of different areas of interest in the resting-state brain network are widespread and affect many networks such as VN, DAN, SN, FPN, and CN. We believe that these functional abnormalities provide new insights into the relationship between depression and network dysfunctions. In the future, these markers could not only help identify depressive symptoms but also serve as targets for appropriate therapies, including pharmacological treatments and functional approaches such as rTMS, which is increasingly used in patients with MDD. Further research is needed, which should focus on creating a common and reproducible method that will allow for an accurate study of the organization of the brain network.

## Figures and Tables

**Table 1:** Demographic and clinical information on the study group.

Demographic Data	Study Group	Control Group
Number of participants	26	26
Mean age (years)	39.4	32.1
Range age (years)	21–64	26–60
Female/male	13/13	16/10
Mean years of education	15.3	15.9
Mean duration of disease (years)	14 (1–44)	-
Antidepressant usage	yes (26)	-
HAM-D score (range)	21.7 (17–32)	-
MADRS score (range)	27.7 (19–41)	-

Abbreviations: HAM-D—Hamilton Depression Scale; MADRAS—Montgomery–Asberg Depression Rating Scale.

**Table 2:** Detailed results of the analysis of intra- and inter-network functional connectivity showing statistically significant results (*p* < 0.05).

Network/Nod 1	Network/Nod 2	Significant Increase (↑) Significant Decrease (↓)
	**Intra-network analysis**	
VN/Occipital	VN/Lateral left	↓
VN/Occipital	VN/Lateral right	↓
VN/Lateral left	VN/Lateral right	↓
	**Inter-network analysis**	
VN/Occipital	DAN/Intraparietal sulcus right	↓
VN/Occipital	SN/Anterior cingulate cortex	↑
VN/Medial	SN/Anterior Insular Cortex right	↑
VN/Medial	SN/Anterior Insular Cortex left	↑
VN/Medial	SN/Anterior cingulate cortex	↑
VN/Medial	SN/Rostral prefrontal cortex right	↑
VN/Lateral left	SN/Supramarginal gyrus left	↑
VN/Lateral left	SN/Supramarginal gyrus right	↑
VN/Lateral left	SN/Anterior Insular Cortex right	↑
VN/Lateral left	SN/Anterior Insular Cortex left	↑
VN/Lateral left	SN/Anterior cingulate cortex	↑
VN/Lateral left	DAN/Intraparietal sulcus left	↑
VN/Lateral right	SN/Supramarginal gyrus left	↑
VN/Lateral right	SN/Supramarginal gyrus right	↑
VN/Lateral right	SN/Anterior Insular Cortex left	↑
VN/Lateral right	SN/Anterior cingulate cortex	↑
VN/Lateral right	SN/Rostral prefrontal cortex right	↑
VN/Lateral right	DAN/Intraparietal sulcus left	↑
SN/Supramarginal gyrus left	CN/Posterior	↓
SN/Supramarginal gyrus right	CN/Anterior	↓
SN/Supramarginal gyrus right	CN/Posterior	↓
SN/Supramarginal gyrus right	FPN/LPFC left	↓
SN/Supramarginal gyrus right	FPN/PPC right	↓
SN/Anterior Insular Cortex right	CN/Posterior	↓
SN/Anterior Insular Cortex right	FPN/LPFC left	↓
SN/Anterior Insular Cortex right	FPN/LPFC right	↓
SN/Anterior cingulate cortex	CN/Anterior	↓
SN/Anterior cingulate cortex	CN/Posterior	↓
SN/Anterior cingulate cortex	FPN/PPC right	↓
SN/Rostral prefrontal cortex right	CN/Posterior	↓
SN/Rostral prefrontal cortex right	FPN/LPFC left	↓
SN/Rostral prefrontal cortex right	FPN/PPC right	↓
SN/Rostral prefrontal cortex right	FPN/LPFC right	↓
SN/Rostral prefrontal cortex left	CN/Posterior	↓
SN/Rostral prefrontal cortex left	FPN/LPFC left	↓
SN/Rostral prefrontal cortex left	FPN/LPFC right	↓

## Data Availability

Data available on request.
